# HelixComplex snail mucus exhibits pro-survival, proliferative and pro-migration effects on mammalian fibroblasts

**DOI:** 10.1038/s41598-018-35816-3

**Published:** 2018-12-05

**Authors:** Claudio Trapella, Roberta Rizzo, Stefania Gallo, Andrea Alogna, Daria Bortolotti, Fabio Casciano, Giorgio Zauli, Paola Secchiero, Rebecca Voltan

**Affiliations:** 1 Department of Chemical and Pharmaceutical Sciences, University of Ferrara, Via Fossato di Mortara 17, 44121 Ferrara, Italy; 2 Department of Medical Sciences, University of Ferrara, Via Luigi Borsari 46, 44121 Ferrara, Italy; 3 Department of Morphology, Surgery, Experimental Medicine and LTTA Centre, University of Ferrara, via Fossato di Mortara 70, 44121 Ferrara, Italy

## Abstract

Snail mucus is a mixture of active substances commonly thought to have healthy properties for the treatment of skin disorders. Although snail mucus is an ingredient of several cosmetic and para-pharmaceutic products, a comprehensive characterization of chemical composition and biological effects is still missing. Crude purified extracts from *Helix aspersa muller* mucus (HelixComplex) were prepared and, after chemical characterization, tested on *in vitro* experimental models. Differently from what expected, HelixComplex was characterized by the presence of small amounts of glycolic acid and allantoin. By using different *in vitro* assays on fibroblast cultures, we found that HelixComplex lacked of cytotoxicity, protected cells from apoptosis (p < 0.05) and, importantly, was able to significantly induce cell proliferation and migration through direct and indirect mechanisms. These effects were associated to morphological changes, cytoskeleton re-organization and release of cytokines. In conclusion, our findings suggest that snail mucus biological effects are attributable to cell proliferation and migration, and pave the way for further investigating snail mucus potential as therapeutic agent.

## Introduction

The snail secretion, or snail mucus, is a mucous substance that covers the entire external surface of the animal and is secreted by particular salivary epidermal glands located at the level of the snail’s foot (pedal glands)^1^. The mucus has different functions for the life of the animal, having adhesive, emollient, moisturizing, lubricating, protective and even reparative properties^2^. To sustain all these biological activities, the snail mucus of *Helix aspersa muller* specie has a complex and still not well characterized composition^1^. The active substances present in this mucus make it a unique natural product not replicable in the laboratory with synthetic chemical compounds.

Since ancient times, the biological properties of *Helix aspersa muller* mucus have been exploited to treat human disorders in particular of the skin, by simply applying the rough mucus. More recently, the use of *Helix aspersa muller* mucus has spread worldwide as constituent of cosmetic products and it has been proposed for the formulation of para-pharmaceutic products for the management of wound^3^ and for the treatment of chronic bronchitis^4^. Although the huge commercial diffusion of products based on *Helix aspersa muller* mucus, a complete description of its chemical composition and the study of its specific biochemical characteristics and biological effects are sustained only by few scientific data^1,5,6^.

Our group has recently developed technologies to collect and purify the *Helix aspersa muller* mucus (Patent N: 10207000117547), referred to as HelixComplex, characterized by a specific and unique molecular profile. Moreover, we have provided a first evidence of biological antimicrobial activity of HelixComplex against a variety of bacteria^7–9^, fungi and viruses (confidential unpublished data). On this basis, the aim of the present study was to further characterize the chemical composition of the HelixComplex and to explore its biological properties in *in vitro* experimental models based on mammalian cells.

## Results

### Chemical and microbiological characterization of snail mucus

**Figure 1:**
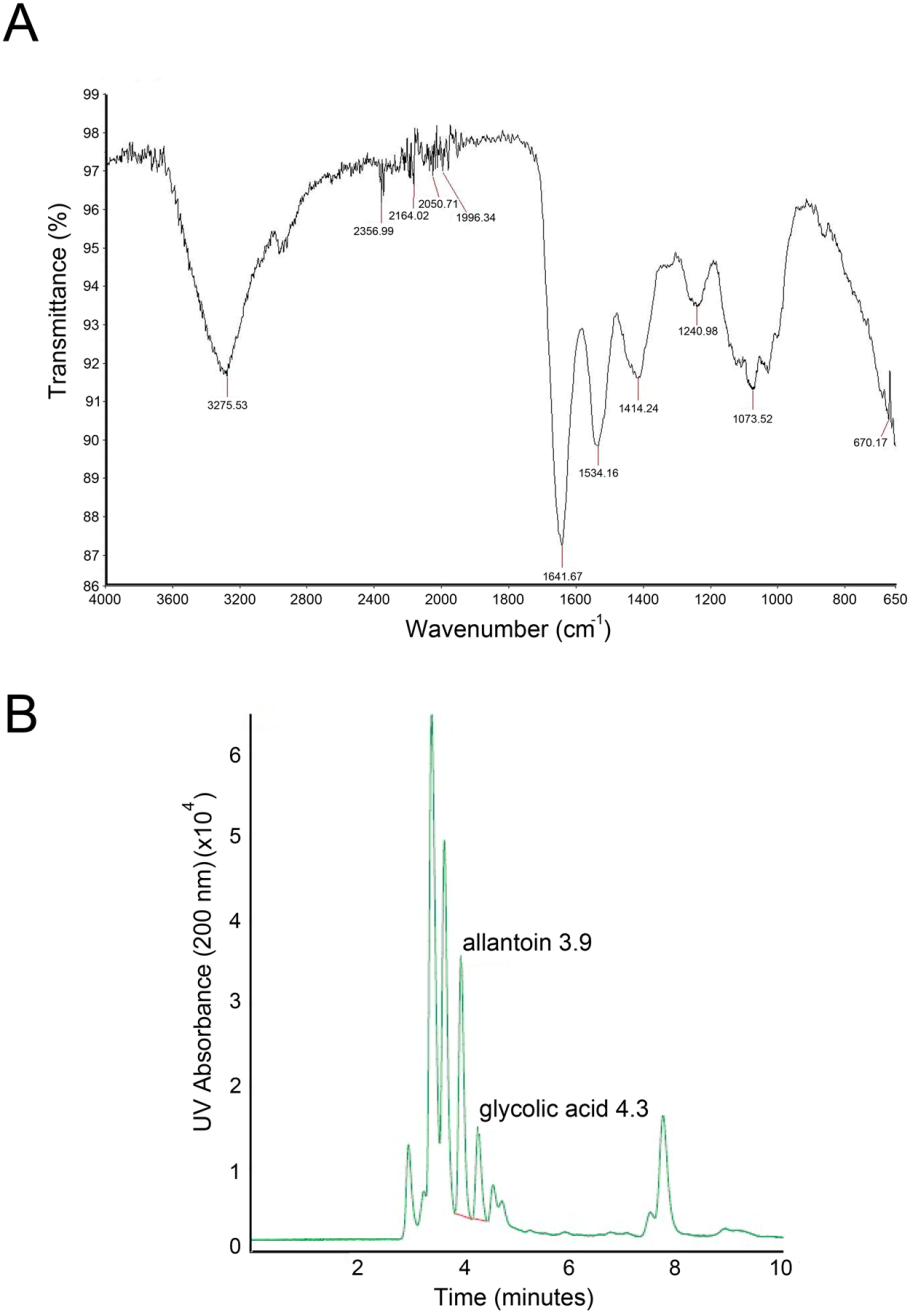
Chemical characterization HelixComplex. In (**A**) infrared spectrum from a representative a HelixComplex sample. In (**B**) HPLC chromatogram of allantoin and glycolic acid from a representative HelixComplex sample.

The characterization of the biological properties of mucus from *Helix aspersa muller* requires the standardization of the purification procedures as well as chemical and microbiological analyses of the extracts. For this purpose, we employed IR-spectroscopy which is utilized in clinical and biological fields^10^. Of note, this technology is efficient for unmixed substances for the determination of the chemical composition through oscillatory behaviors of excited bonds by a light source. Since snail mucus is a complex mixture of substances, we focused on some specific areas of the spectrum. As shown in Fig. 1A, the IR spectra of the HelixComplex is unique and allowed us to obtain information about the quality of the extract: the absorbance peak to 3250 cm^−1^ is typical of hydroxylic groups of hydrophilic amino acids; the area between 3000 and 3200 cm^−1^ defined aromatic overtone, due of aromatic amino acids; whereas, peaks at 1645cm^−1^ and at 1540 cm^−1^ are the most important because they are typical of amide bond, thus indicating the presence of proteins.

Table 1 reports the main qualitative-quantitative properties and composition of HelixComplex identified by chemical analyses. Of note, elution of allantoin and glycolic acid was observed in our *Helix aspersa muller* extracts only after 3.9 ± 0.1 and after 4.3 ± 0.1 minutes, respectively (Fig. 1B), indicating a lower concentration than expected from what is usually reported in snail mucus commercial preparations.

**Table 1:** Qualitative-quantitative chemical and microbiological analysis of HelixComplex.

Specification	Values	Measure unit
Aspect	Clear	
Color	Light yellow	
Smell	Odourless	
pH	6.0–7.0	
Density	1–1.1	
Dry residual	2-3	g/L
Yield%	0.1-0.2	
Minerals	250–350	mg/L
Heavy metals	Absent	
Proteins	100–250	mg/L
GAGs (sulfurated)	29–90	mg/L
GAGs (unsulfurated)	70–80	mg/L
Glycolic acid	<200	mg/L
Allantoin	<20	mg/L
Poliphenols	70–80	mg/L
Sugars	0.010–0.027	g/L
Collagen	1–100	mg/L
Gram +	0	CFU
Gram −	0	CFU
Fungi	0	CFU

Finally, the microbial characterization (Table 1) of HelixComplex confirmed that the sterilizing filtration process after the purification procedure (Patent N: 10207000117547) was able to sterilize the product by removing all the bacterial and fungal contaminations without the addition of any preservative. Moreover, we observed that the HelixComplex can be stored at −80/+4 °C for over 12 months remaining microbiologically pure and maintaining unaltered its biological effects (data not shown).

### Lack of cytotoxicity of HelixComplex

**Figure 2:**
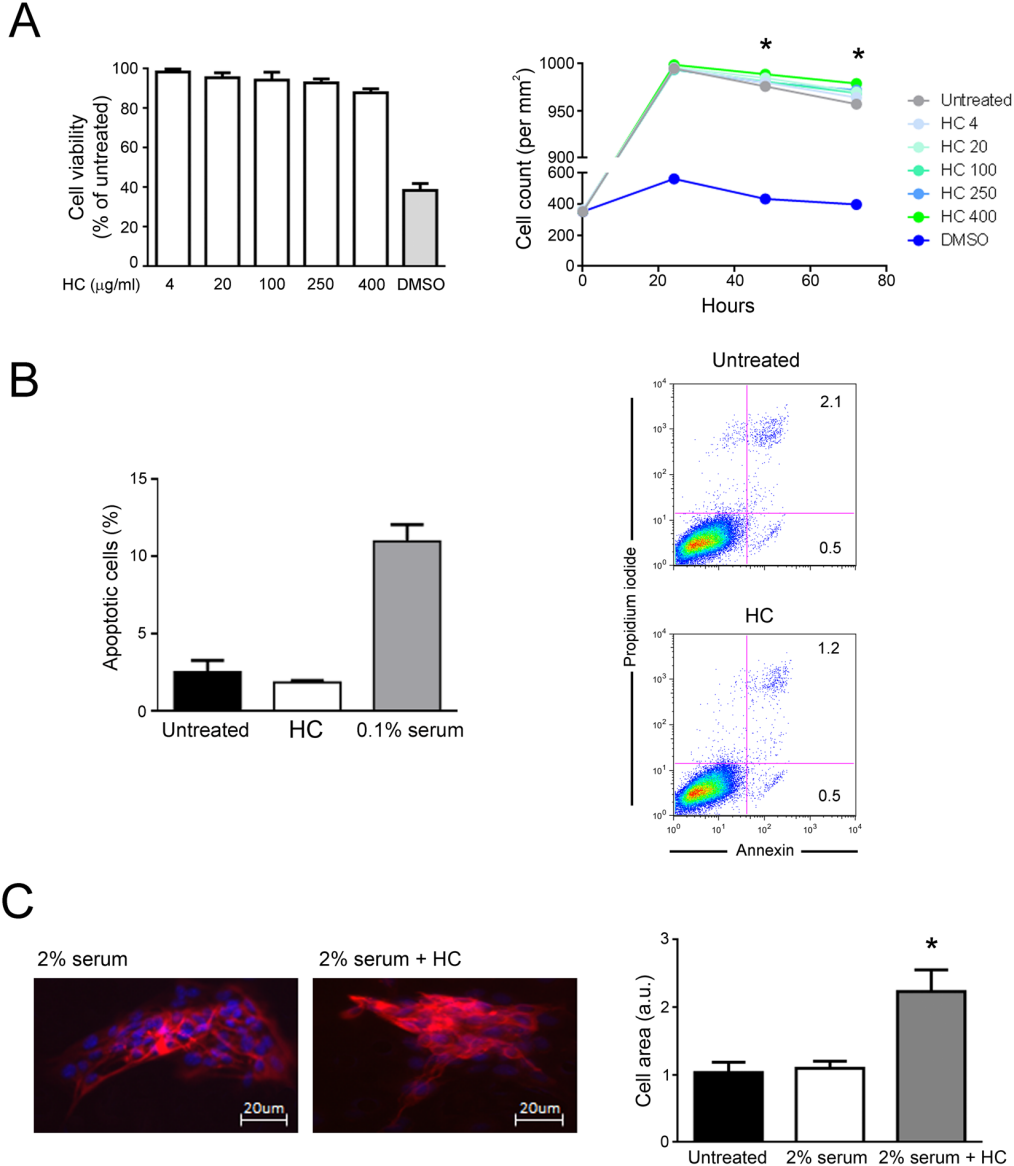
Lack of cell cytotoxicity by HelixComplex treatment. In (**A**) fibroblasts were exposed to increasing doses of HelixComplex for analysis of cell viability (from 4 µg/ml to 400 µg/ml). Left panel: cell viability, examined by MTT colorimetric assays, was calculated at 24 hours as percentage with respect to the untreated cultures (set to 100%). Right panel: cell number was monitored over time for up to 72 hours, starting from a high density cell culture. DMSO (10%) was used as positive control of cell death. In (**B**) apoptosis was evaluated on fibroblasts treated for 48 hours with a high dose of HelixComplex (400 µg/ml) and calculated as percentage of Annexin V/PI double positive cells on the total population for each treatment. Representative plots of apoptotic cells analyzed by flow-cytometry are shown. In (**C**) fibroblasts were cultured for 48 hours in medium with 2% serum with or without 400 µg/ml HelixComplex. Medium with 10% serum was used in the untreated control (Untreated). Left panel: representative immunofluorescence images of actin organization (red staining). Nuclei were colored with DAPI (blue staining). Right panel: cell surface was measured and reported in arbitrary units (AU). In (**A**–**C**) data are reported as the mean ± SD of results from at least three independent experiments. The asterisk indicates p < 0.05 respect to untreated cultures. HC: HelixComplex.

To evaluate the biological effects of the HelixComplex, in a first set of experiments fibroblasts were treated *in vitro* with different concentrations (4–400 µg/ml) of mucus, for analysis of the impact on normal cell viability and morphology. As shown in Fig. 2A,B, lack of cytotoxicity and of any cytostatic effect was observed in high density cultures at all tested concentrations. Of note, a significant increase in cell number was registered with the 400 µg/ml dose at 48 and 72 hours after the treatment in comparison with untreated samples (p < 0.05), suggesting that HelixComplex was able to counteract the inhibition of proliferation due to the accomplishment of *in vitro* confluence. In addition, treated fibroblasts exhibited significant morphological changes associated with modulation of cytoskeleton organization and cell enlargement (p < 0.05), further confirming lack of cytotoxicity and suggesting a possible effect on cellular motility (Fig. 2C).

### HelixComplex promotes fibroblasts proliferation

**Figure 3:**
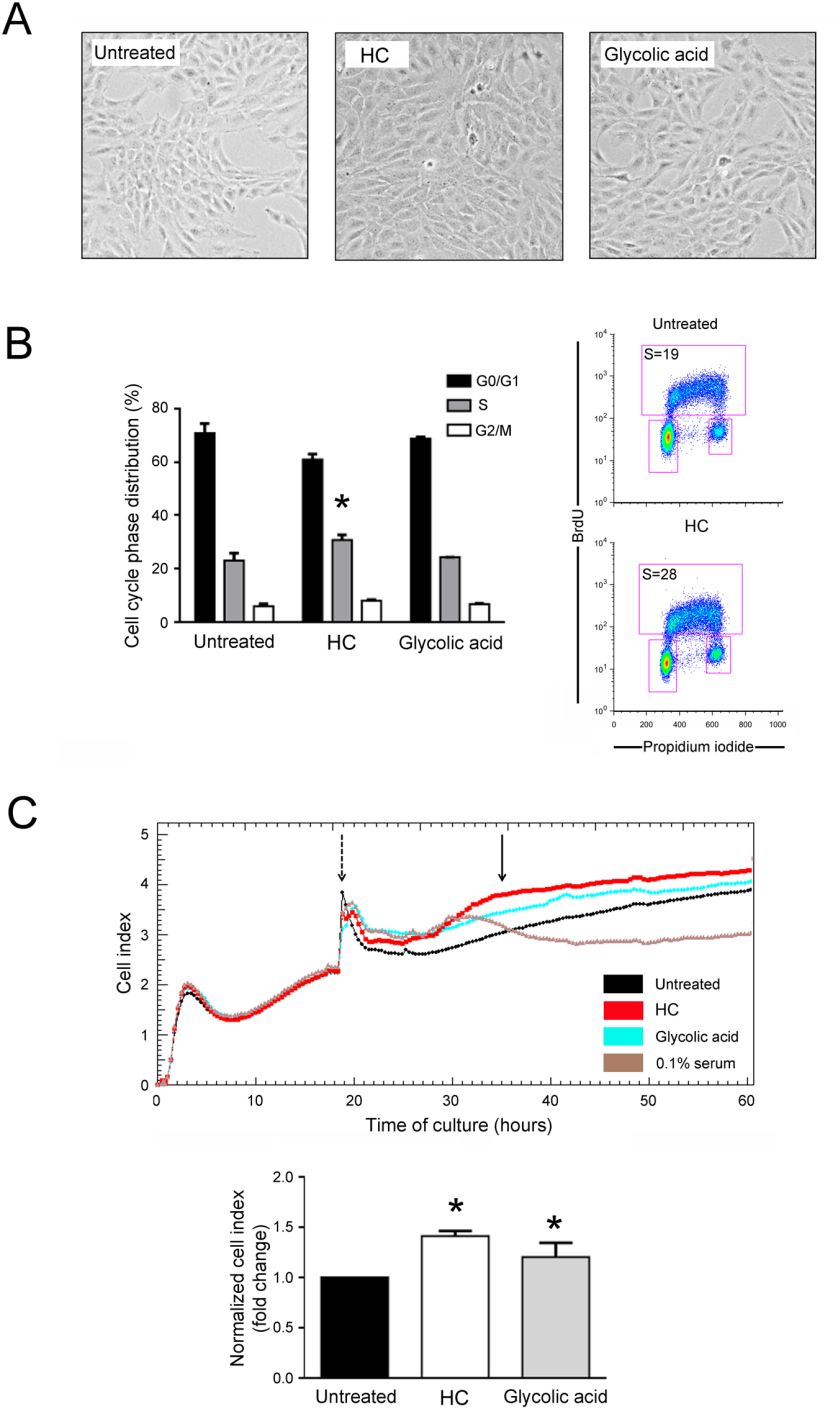
HelixComplex promotes cellular proliferation. Cultures of fibroblasts were exposed to high dose of HelixComplex (400 µg/ml) for up to 48 hours. In (**A**) representative images taken by light microscopy of monolayers of fibroblasts untreated or treated with HelixComplex or glycolic acid (0.1 mM) at 48 hours. Magnification 100x. In (**B**) cell distribution in the different phases of the cell cycle was calculated from the flow cytometric dot plots after BrdU/PI staining of cultures after 36 hours of treatment and was expressed as percentage of the total population. Representative flow cytometric dot plots of cell-cycle profiles are shown: the rectangles represent the cells in the different (G0/G1, S, G2/M) phases of the cell cycle and the percentage of cells in S-phase is indicated for each treatment. In (**C**) fibroblasts were treated with HelixComplex or glycolic acid and monitored for proliferation with the xCELLigence system. Upper panel: representative plot of Cell index of fibroblasts treated with HelixComplex or glycolic acid. Fibroblasts grown in starvation medium (0.1% serum) were used as negative control of proliferation. Dashed arrow indicates the time in which treatments were added to cell cultures. Arrow indicates the time in which Cell index was quantified. Lower panel: Cell index quantification at 16 hours after treatment (normalized relative to 0 hour) and expressed as fold of modulation with respect to untreated cultures set at 1. In (**B**,**C**) data are reported as the mean ± SD of results from at least three independent experiments. The asterisk indicates p < 0.05 respect to untreated cultures. HC: HelixComplex.

To investigate the possible role of HelixComplex on promoting cell proliferation, a second panel of experiments was performed by using multiple experimental approaches (Fig. 3).

At microscopic examination of low density seeded cell cultures, fibroblast monolayers treated with HelixComplex (400 µg/ml) reached a full confluence after 48 hours, while, at the same time point, untreated cultures still appeared as sub-confluent monolayers (Fig. 3A). Moreover, cell cycle analysis by BrDU incorporation and flow cytometry revealed that treatment with HelixComplex was able to modify the cell cycle phase distribution, inducing a significant (p < 0.05) increase in the percentage of cells in the S phase (36 hours) (Fig. 3B). Finally, the ability to promote cell proliferation was finely assessed by using a real-time analysis based on the xCELLigence technology (Roche). As shown in Fig. 3C, at the time points examined, the HelixComplex (400 µg/ml) induced a significantly higher fibroblast proliferation compared to untreated cultures (p < 0.05) and remained higher over 24/48 hours of treatment until monolayer confluence.

In parallel, fibroblasts were cultivated with glycolic acid (0.1 mM) used as positive control for its known ability to induce cell proliferation^11^. Interestingly, the effects induced by the HelixComplex in terms of fibroblast proliferation were comparable, or even higher, than those induced by glycolic acid (Fig. 3A–C).

### HelixComplex exhibits protection from apoptosis

**Figure 4:**
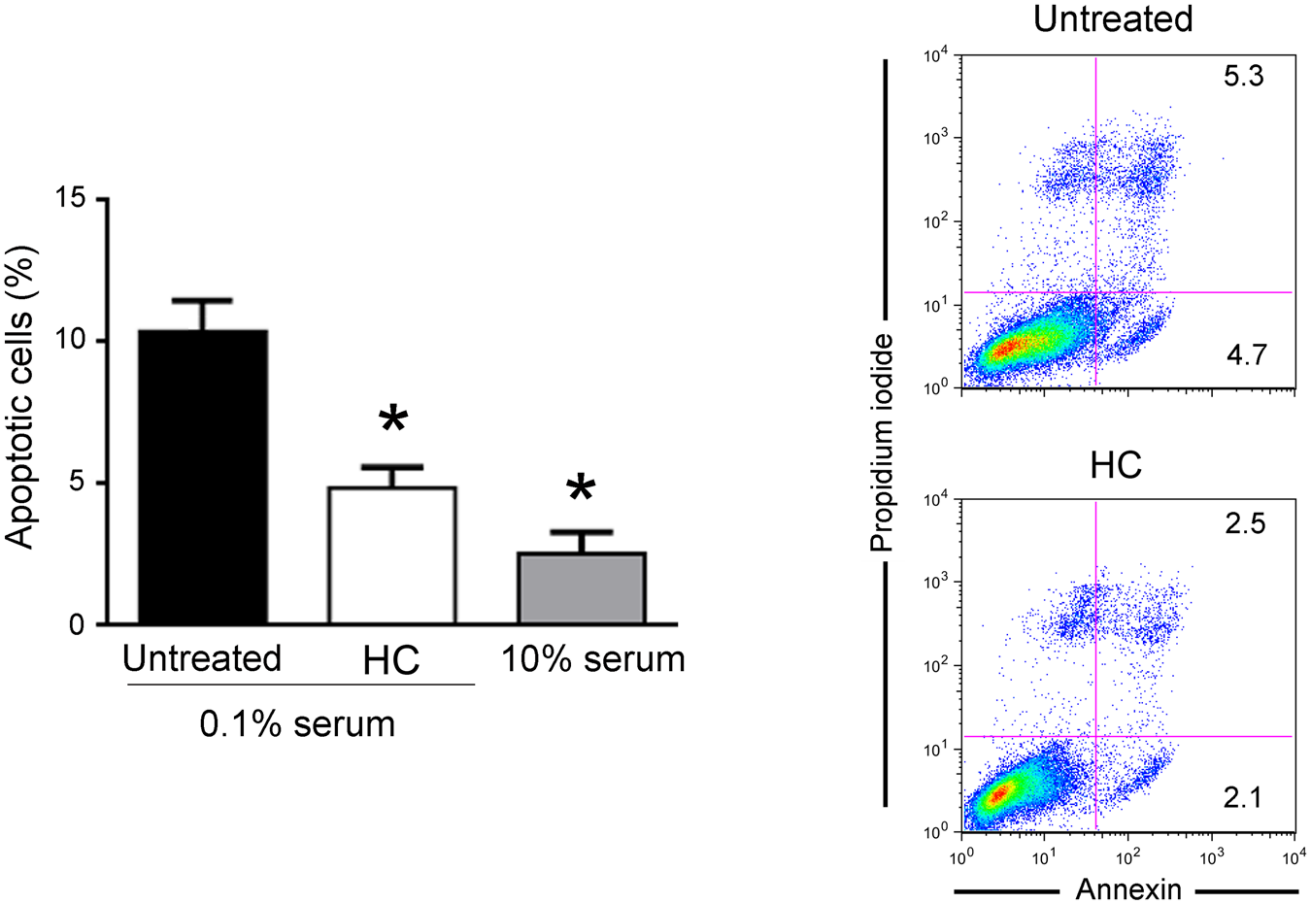
HelixComplex exhibits protection from apoptosis. Fibroblasts were grown in starvation medium (0.1% serum) and treated with HelixComplex (400 µg/ml). Fibroblasts grown with standard medium (10% serum) were used as negative control of apoptosis induction. The induction of apoptosis was calculated after 48 hours of treatment as percentage of Annexin V/PI double positive cells on the total population for each treatment. Data are reported as the mean ± SD of results from at least three independent experiments. The asterisk indicates p < 0.05 respect to untreated cultures. Representative plots of apoptotic cells analyzed by flow-cytometry are shown. HC: HelixComplex.

To further characterize the effects of mucus on fibroblast biology, next experiments were performed on cells grown with medium containing a low percentage of serum (0.1%), a condition that mimics a physiological stress that reduces the income of nutrients to a tissue (serum starvation). As shown in Fig. 4, in cultures grown with low serum (0.1%), fibroblasts were triggered to dead by apoptotic process, as assessed by PI incorporation and flow cytometry analysis. Of note, in this culture setting, the exposure of fibroblasts to HelixComplex significantly (p < 0.05) reduced the percentage of apoptosis level induced by serum starvation (Fig. 4). Taken together, our data indicate that the increase of cell proliferation by HelixComplex treatment due to cell cycle induction is coupled with apoptosis protection.

### HelixComplex promotes cell migration and wound repair

**Figure 5:**
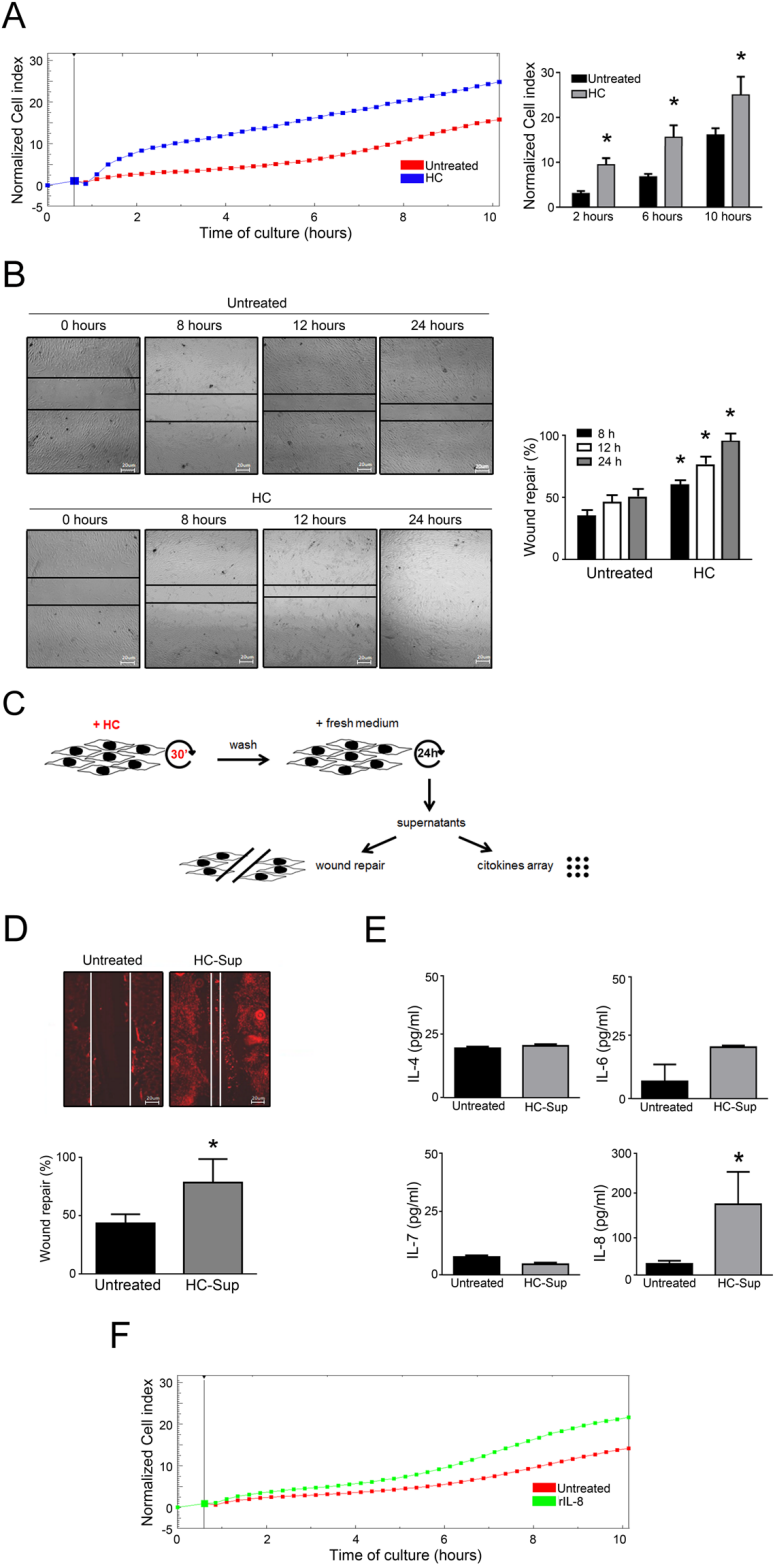
HelixComplex promotes cell migration and wound repair. Dynamic monitoring of fibroblasts migration in response to HelixComplex treatment (400 µg/ml) by xCELLigence and scratch assays. In (**A**) fibroblasts were seeded into the upper chamber and monitored for migration through the lower chamber in response to HelixComplex with the xCELLigence system. Left panel: a representative plot of Cell index (normalized to the first reading of the plate) of migrated fibroblasts is shown. Right panel: Cell index quantification at different time points after the beginning of migration (normalized to the first reading). In (**B**) scratch-wound healing assay. Left panel: representative images taken at the indicated time points post wounding are displayed. Right panel: quantification of wound repair at the indicated time points expressed as percentage of wound repair in comparison with the 0-hour time point. In (**C**) a schematic representation of the experiments performed with supernatants of HelixComplex shortly-exposed fibroblasts. In (**D**) scratched fibroblasts were treated with supernatants from fibroblasts cultures shortly exposed to HelixComplex (HC-Sup) and observed 24 hours after wounding. Upper panel: immunofluorescence images of actively migrating fibroblasts stained for endogenous actin (red staining). Lines show the extent of the wound closure. Scale bars: 20 μm. Bottom panel: quantification of wound repair expressed as percentage of repair in comparison with the 0-hour time point. In (**E**) histograms represent cytokines detectable in supernatants of fibroblast 24 hours after short-exposure to HelixComplex. In (**F**) fibroblasts were seeded into the upper chamber and monitored for migration through the lower chamber in response to recombinant human IL-8 (rIL-8) with the xCELLigence system; a representative plot of Cell index (normalized to the first reading of the plate) of migrated fibroblasts is shown. Data are reported as the mean ± SD of results from at least three independent experiments. The asterisk indicates p < 0.05 respect to untreated cultures. HC: HelixComplex.

In the next experiments, we have explored the potential effects of HelixComplex on cell motility. The results of migration experiment performed by using the xCELLigence real-time analyzer clearly indicated that fibroblasts responded to HelixComplex as attractant stimulus in a time-dependent manner inducing cell migration response more efficiently (p < 0.05) than control (Fig. 5A). Considering that fibroblast migration is a relevant process occurring during the wound repair, we next performed classical scratch assays to further evaluate the wound healing potential of HelixComplex (Fig. 5B). Data obtained from these experiments reinforced the migration data, showing a faster closure of the scratch, with a significant (p < 0.05) higher number of cells present into the scratch area in cultures exposed to HelixComplex (400 µg/ml) with respect to control cultures (Fig. 5B).

In additional experiments, represented in Fig. 5C–E, fibroblast cultures were pre-exposed to the HelixComplex mucus for 30 minutes, washed with PBS and grown for additional 24 hours with normal fresh medium (without additional treatment). Then, the supernatants from these cultures were added to new scratched fibroblast cultures for wound healing assays and in parallel analyzed for secreted cytokines content, since fibroblasts are known to secrete cytokines involved in wound repair^12,13^.

The scratch closure was examined 24 hours after the treatment and revealed that the supernatants of fibroblasts pre-exposed for a short time to HelixComplex were able to induce a scratch closure (p < 0.05 respect to untreated) (Fig. 5D) in a similar fashion to what observed in fibroblasts directly treated with HelixComplex for a longer period (Fig. 5B).

At the same time, the cytokine analysis of the same supernatants revealed that, among the examined panel of cytokines (including IL-1α, IL-1β, IL-2, IL-4, IL-6, IL-7, IL-8, IL-10, IL-12, GM-CSF, IFN-γ and TNFα), only IL-4, IL-6, IL-7 and IL-8 were detectable and IL-8 specific release was significantly (p < 0.05) higher respect to the background from fibroblasts treated with control supernatants (Fig. 5E). To confirm that secreted IL-8 could have an effect on fibroblast migration during wound repair, in the last set of experiments we used recombinant IL-8 (in the same range of concentration observed in the supernatants) as chemoattractant and observed that fibroblast migrated toward it in time-depending manner (Fig. 5F).

## Discussion

In the present study, we provided a detailed description of the molecular composition of the *Helix aspersa muller* mucus HelixComplex, that we previously showed characterized by anti-bacterial properties^7^. Moreover, we characterized its biological effects on mammalian cells, demonstrating for the first time pro-survival, pro-proliferation and pro-migration effects in *in vitro* experiments of wound healing settled with mammalian fibroblasts.

The results obtained from the HelixComplex chemical analyses defined its unique molecular features as reported in Table 1. The chemical composition of the snail mucus extract was determined using colorimetric, UV and IR analyses, due to the high complexity of the mixture composed by small- and macro- molecules, as observed by Liquid Chromatography Mass Spectrometry (LC/MS) and Liquid Chromatography Tandem Mass Spectrometry (LC/MS/MS) analyses (Supplementary Figs 1 and 2).

Unexpectedly, in all analyzed batch we observed only small amounts of two components that were previously considered the signature molecules of snail mucus and essential for its biological/curative effects^3^, glycolic acid and allantoin. This observation ruled out the possibility that the biological effects that we observed on mammalian fibroblasts were simply due to these compounds, and suggested the implication of other additional active molecules. This hypothesis was supported by the observation that the effects on fibroblasts proliferation were significantly triggered by treatment with HelixComplex, while glycolic acid, used as control, showed lower proliferative capacity. The crude mucus composition certainly deserves further investigation to elucidate in depth the chemical structure of the still unknown molecules and to possibly identify some specific biomarkers.

In fact, a synergist activity of several molecules could justify the HelixComplex effects on inducing proliferation and migration of fibroblasts during *in vitro* wound repair and might be at the basis of the efficient induction of wound repair observed upon the use of para-pharmaceutical products containing snail mucus. In our first experimental setting of wound healing, that implied exposure of fibroblasts to HelixComplex for 24 hours, we demonstrated pro-motility effects on fibroblasts supported by real-time migration assays and classical scratch assays showing a significant healing reparative effects. Similar effects were evident also on a second setting of wound healing that implied treatment of fibroblasts with supernatant collected from cultures pre-exposed shortly (30 minutes) to HelixComplex. This interesting result suggested a conceivable ability of HelixComplex to induce the release from the treated fibroblasts of soluble molecules with autocrine and paracrine effects. In line with this, by analyzing of the cytokines content of the same supernatants of shortly pre-exposed fibroblasts, we demonstrated that HelixComplex treatment induced a significant secretion of IL-8. We believe that IL-8 had an important role in the observed effects, since it has recognized chemotactic effects on several cell types during inflammation events (such as acute or chronic wound)^14–17^. In line with this, we demonstrated IL-8 activity as chemo-attractant factor also in migration assays.

Overall, our results suggest that HelixComplex promoted cell migration and wound healing process both directly (through compounds present in the purified extract) and indirectly, by inducing the release from treated cells of IL-8 together with other still unidentified soluble factors. Of note, all the experiments have been performed on both human and murine fibroblasts, and no significant differences between the two models have been observed.

Considering that the biological activity of the HelixComplex, such as any other biological material, could change with time due to deterioration, we studied the stability of the product over time and observed that the effects on proliferation, migration and wound healing remained unaltered after long storage period (9 months) and several freezing (−80 °C)/thawing processes (data not shown). This observation will be useful for the potential use of HelixComplex as natural product, as well as active component of industrial products.

Although our data would be reinforced by *in vivo* experiments further investigating the biological effects of HelixComplex, we believe that our results offer for the first time a scientific reason and an opportunity for a potential therapeutic use of HelixComplex in clinical relevant pathological situations, such as chronic wounds.

## Methods

### *Helix aspersa muller* mucus collection and sterilization

*Helix aspersa muller* mucus (HelixComplex) was collected in an Italian fosterage. Since the conventional use of NaCl to induce mucus production deeply affects protein content and consequently mucus quality, we have standardized an extraction method with the use of low concentrations of NaCl (3%) and an extractor machine (Beatrix; Colognesi industries; Ferrara, Italy) that collects about 600 ml of crude extract from 500 snails (about 10 kg) after 45 minutes (Patent N WO2013011371A1). Mucus was than sterilized with a peristaltic pump and a filtration device (0.2 µm; Pall) specifically developed for mucus filtration (Patent N 10207000117547) and then stored at 4 °C or −80 °C.

### Chemical characterization

The crude extract from different batches of HelixComplex was chemically analyzed using standard analytical techniques such as infrared spectrometry (IR) and HPLC analysis to evaluate the protein quality and the allantoin and glycolic acid content, respectively^18^. In order to obtain only the dry part, samples were frozen in liquid nitrogen and lyophilized overnight to obtain a solid powder that was then subjected to the infrared analysis with a Spectrum 100 (Perkin Elmer, Waltham, MA, USA) equipped with a ZnSe diamond to obtain a qualitative determination of the total protein content and to a Bradford assay (Bio-Rad, Hercules, CA, USA) to evaluate the protein amount.

The mineralization of the lyophilized samples was performed and the presence of metals was measured by spectroscopy of atomic absorption. To this purpose, 7 ml of 70% HNO_3_ were added to the samples in a test tube with a refrigerant, in order to condense the vapors formed during the mineralization process. The tubes were placed in the mineralizator for 20 minutes at 50, 90, 140 °C and for 40 minutes at 200 °C. At the end of the mineralization process, all organic molecules were oxidized to H_2_O and CO_2_, whereas all metals converted in soluble nitrates salts. After the system was chilled, samples were added with 1 ml of 40% H_2_O_2_ to complete the oxidation of the organic matter, and then held for 20 minutes at 200 °C (stripping process). Finally, the sample was recovered with milliQ water in a 20 ml flask filtered with a paper filter Whatman and analyzed using atomic adsorption instrument (Perkin Elmer 1100B) employing air/acetylene flame.

Qualitative and quantitative analyses of specific chemical elements are reported in Supplementary Methods.

### Microbiological characterization

To test the eventual microbiological contamination, 100 µl of HelixComplex were plated on culture dishes containing culture media Tryptic Soy agar (TSA) (Biomerieux, Italy). The number of colonies was evaluated after 24–48 hours at 37 °C and expressed as colony forming unit (CFU). The identification of contaminating bacteria was performed by Gram staining (Liofilchem, Italy). The presence of contaminating fungi was evaluated by plating HelixComplex on Sabouraud medium plates (Biomerieux, Italy).

### Cell cultures and treatments

Human dermal fibroblasts (MRC-5) and murine embryo fibroblasts (NIH-3T3) were purchased from Lonza and grown in EMEM and DMEM, respectively, containing 10% FBS, pen/strep and L-Glut (Lonza, Walkersville, MD). Cell cultures were maintained at 37 °C in a humidified atmosphere with 5% CO_2_. Cell cultures were analyzed for cell shape and growth changes and phase-contrast images were recorded with EVOS digital inverted microscope (Advanced Microscopy Group, Bothell, WA).

HelixComplex preparations were used for *in vitro* treatments of cell cultures using a range of concentrations previously determined in dose-response assays (4–400 μg/ml). Glycolic acid was used as fibroblast proliferation-inducer positive control at the concentration of 0.1 mM^11^. For growth and proliferation assays, fibroblasts were seeded and treated when reached 50–60% of confluence (using a plating density of 10^4^ cells/ml).

### Assessment of cell viability, cell cycle profile and apoptosis

At different time points after treatment, cell viability was examined by Trypan blue dye exclusion and MTT (3-(4,5-dimethilthiazol-2yl)-2,5-diphenyl tetrazolium bromide) colorimetric assay (Roche Diagnostics Corporation, Indianapolis, IN) for data confirmation, as previously described^19^. The cell cycle profile was analyzed by incubating the cells with 50 μmol/L 5-bromodeoxyuridine (BrdU; Sigma, St Louis, MO) at 37 °C for 1 hour. Anti-BrdU antibody (Ab) was bound to BrdU incorporated into neosynthesized DNA, and the complex was detected by fluorescein isothiocyanate-conjugated secondary Ab. Cells were then counterstained with propidium iodide (PI; 50 μg/ml) and analyzed by flow cytometry. The amount of apoptosis was quantified by Annexin V-FITC/PI staining (Beckman Coulter Inc., Brea, CA) using a FACSCalibur flow cytometer (BD Biosciences, San José, CA). To avoid non-specific fluorescence from dead cells, live cells were gated tightly using forward and side scatter, as described^20^. Adherent cells were recovered with 0.25% trypsin-EDTA and pooled with floating cells to analyze the degree of cell death and apoptosis in the entire cell population. In selected experiments, cells were treated in starvation conditions using reduced serum concentration (0–2%).

### Assessment of cell proliferation and migration

Cell proliferation and migration analyses were performed using the xCELLigence real time cell analyzer DP-RTCA (Roche Diagnostics, Mannheim, Germany), which records changes in impedance (reported as a Cell index, CI) over time in a non-invasive system, as previously detailed^21^. Briefly, for the proliferation assay, the background impedance was performed using RTCA DP E-Plates 16 following the standard protocol provided in the software with 100 µl of complete medium. Fibroblasts were seeded in quadruplicate at three different concentrations with 50 µl of complete medium and left to equilibrate at room temperature for 30 minutes before starting the measuring. Cells were allowed to adhere and proliferate overnight at 37 °C in a humidified atmosphere with 5% CO_2_ until pre-established CI before treatments (50 µl). The CI of the proliferating cells was recorded up to 72 hours.

Migration experiments were performed using RTCA DP CIM-Plates 16. Fibroblasts were seeded in the upper chamber in quadruplicate at three different concentrations and left to equilibrate at room temperature for 30 minutes. Migration kinetics were analyzed in the absence or presence of HelixComplex (400 µg/ml) or controls, such as serum or rIL-8 (1 ng/ml; R&D Systems, Minneapolis, MN) in the bottom chamber and recorded up to 72 hours. Data were analyzed using the xCELLigence software (Roche, version 1.2.1) and expressed as mean ± SD of CI normalized to the last CI recorded before the time of cells treatment.

For scratch assays, fibroblasts were seeded at the final concentration of 1 × 10^6^ in a 6-well plate. After 24 hours, medium was removed and a linear scratch in the middle of the well was done using a p200 tip. Then, fresh medium with 2% serum with or without HelixComplex (400 µg/ml) was added to each well. Wells were checked for scratch repair by optical microscopy.

### Immunofluorescence

Immunofluorescence for the detection of cytoskeleton organization was performed with an anti-actin mouse monoclonal antibody-PE (mAb) (Santa Cruz Biotechnology, Santa Cruz, CA), as previously described^22^. Cell area was measured on digital pictures of the cells using the NIS-Elements D software (Nikon Instruments Europe, Firenze, Italy). DAPI (ThermoFisher Scientific, Waltham, MS, USA) staining was used for nuclei detection.

### Cytokine analyses

Fibroblast cultures were pre-exposed to the HelixComplex (400 µg/ml), or left untreated, for 30 minutes, washed with PBS and grown for additional 24 hours with normal fresh medium. Then, culture supernatants were collected and analyzed for a panel of cytokine (IL-1α, IL-1β, IL-2, IL-4, IL-6, IL-7, IL-8, IL-10, IL-12, GM-CSF, IFN-γ and TNFα) using Ciraplex Assays (Aushon, MA, USA), according to the manufacturer’s instructions.

### Statistical analyses

Statistical analysis has been conducted using the Stat View software package (SAS Institute Inc, Cary, NC, US). The data have been analyzed by Student t-test. Statistical significance was assumed for p < 0.05 (two tailed).

## Electronic supplementary material

Supplementary Information

## Data Availability

All authors confirmed the availability of data and materials upon request.
