# Author Correction: HelixComplex snail mucus exhibits pro-survival, proliferative and pro-migration effects on mammalian fibroblasts

**DOI:** 10.1038/s41598-020-58750-9

**Published:** 2020-01-30

**Authors:** Claudio Trapella, Roberta Rizzo, Stefania Gallo, Andrea Alogna, Daria Bortolotti, Fabio Casciano, Giorgio Zauli, Paola Secchiero, Rebecca Voltan

**Affiliations:** 1 Department of Chemical and Pharmaceutical Sciences, University of Ferrara, Via Fossato di Mortara 17, 44121 Ferrara, Italy; 2 Department of Medical Sciences, University of Ferrara, Via Luigi Borsari 46, 44121 Ferrara, Italy; 3 Department of Morphology, Surgery, Experimental Medicine and LTTA Centre, University of Ferrara, via Fossato di Mortara 70, 44121 Ferrara, Italy

Correction to: *Scientific Reports* 10.1038/s41598-018-35816-3, published online 05 December 2018

This Article contains an error in Figure 5b, where an incorrect image was used for HelixComplex treated samples at 0 hours. The correct Figure 5b appears below as Fig. 1.

**Figure 1:**
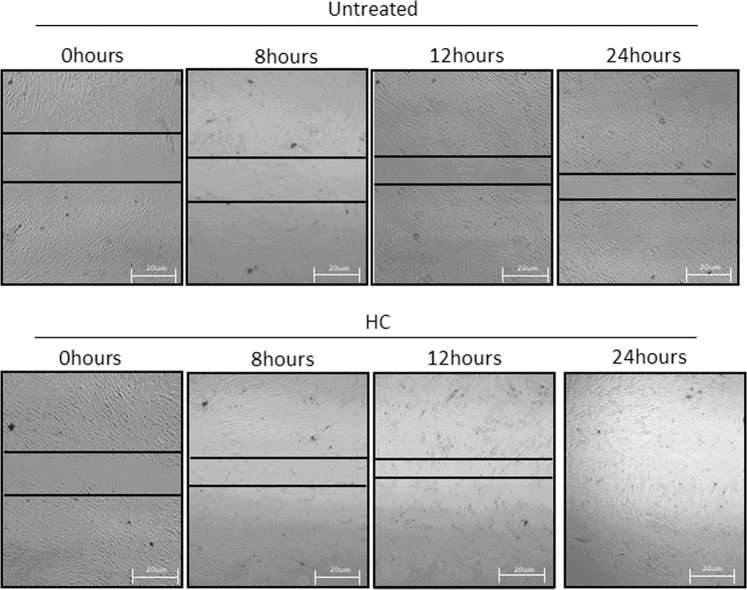
.

