# Supplementary material for: HelixComplex snail mucus exhibits pro-survival, proliferative and pro-migration effects on mammalian fibroblasts
Source: Sci Rep. 2018 Dec 5;8:17665. doi: 10.1038/s41598-018-35816-3 (PMC6281574; doi:10.1038/s41598-018-35816-3)
Supplement: Supplementary file 1 — Supplementary Information [file 41598_2018_35816_MOESM1_ESM.docx]

**HelixComplex snail mucus exhibits pro-survival, proliferative and pro-migration**

**effects on mammalian fibroblasts**

Claudio Trapella^1^, Roberta Rizzo^2^, Stefania Gallo^3^, Andrea Alogna^1^, Daria Bortolotti^2^,

Fabio Casciano^3^, Giorgio Zauli^3^, Paola Secchiero^3^ and Rebecca Voltan^3^

^1^Department of Chemical and Pharmaceutical Sciences, University of Ferrara, Via Fossato di Mortara 17, 44121 Ferrara Italy

^2^Department of Medical Sciences, University of Ferrara, Via Luigi Borsari 46, 44121 Ferrara, Italy

^3^Department of Morphology, Surgery, Experimental Medicine and LTTA Centre, University of Ferrara, via Fossato di Mortara 70, 44121 Ferrara, Italy

**Supplementary Methods**

**Qualitative and quantitative analysis of specific chemical elements of HelixComplex**

Allantoin and glycolic acid concentrations were simultaneously determined by HPLC analysis using a Beckman System Gold 125 instrument coupled with a Beckman 166 UV detector set at 200nm and equipped with a Synergy Hydro-RP C-18 Column (250mm x 4.6mm I.D., 5μm particle size Phenomenex, Torrance, CA, USA) [18]. The mobile phase was a potassium phosphate solution (KH_2_PO_4_, pH 2.9; 10mM) and acetonitrile (CH_3_CN) with a flow of 0.7 ml/min at 30°C. Elution was performed with isocratic of solution A (KH_2_PO_4_) for 10 minutes and reduced at 30% in 5 minutes. This percentage was maintained for 10 minutes for the perfect clean-up of the column. Then the percentage of solution A was restored at 100% in 10 minutes and maintained for other 10 minutes for the reconditioning of the column before inject the next sample. The standard curve was prepared in triplicate dissolving increasing amount of allantoin (Fluka, Milan, Italy) and of glycolic acid (Sigma-Aldrich, Milan, Italy) in phosphate buffer.

Spectrophotometric assay was used to quantify sulfated and non-sulfated glycosaminoglycans (GAGs). The content of sulfated GAGs was determined using the DMMB (1,9-Dimethyl-Methylene Blue, Sigma-Aldrich) assay according to the manufacturer’s instructions. Standards for GAGs quantification (chondroitin-sulfate, heparan-sulfate and dermatan-sulfate, Sigma-Aldrich, Milan, Italy) were prepared in 100 mM ammonium acetate in a range of concentrations 0-50 µg/ml. The measure was performed in duplicate in a microplate reader (Infinite 200 PRO Series Multimode Reader from Tecan Trading AG) at 525nm.

The amount of total phenols, generally related with the antioxidant activity of a sample, was measured with the Folin-Ciocalteou (F-C) assay. Briefly, 0,5g of the standard polyphenol gallic acid (Sigma Aldrich) were dissolved in 10% ethanol. In a cuvette 20µl of the sample were mixed with 1,58ml of deionized water, 100µl of F-C reagent and 300µl of 20% Na_2_CO_3_ solution. The analysis was performed after 90 minutes in a dark field. The total phenolic content was calculated from the calibration curve, obtained with increasing concentrations of gallic acid and measured with a spectrophotometer (Beckman DU 520) at 765nm.

Free sugars (D-glucose and D-fructose) and disaccharides (sucrose) were analyzed with the D-glucose D-fructose Kit (Megazyme inc, USA). D-glucose concentration was determined before and after sucrose hydrolysis with the β-fructosidase enzyme. The D-fructose amount was achieved as consequence of D-glucose quantification, after isomerization caused by the phosphoglucose isomerase.

Quantification of collagen was performed using a colorimetric method for hydroxyproline analysis. Briefly, a standard curve with increasing amount of hydroxyproline was prepared. Mucus sample was dissolved in 100µl of concentrated hydrochloric acid for the release of the hydroxyproline aminoacid after complete protein hydrolysis. Hydroxyproline was quantified using the oxidation buffer citrate/acetate and DMAB.

**LC/MS analysis of crude extract of snail mucus**

The crude lyophilized extract of snail mucus (0.5mg) was dissolved in 1.5ml of a 60% acetonitrile, 40% water and 0.1% formic acid solution and filtered off to a 0.22μm regenerate cellulose filter. The clear solution was then analyzed with ESI-Q-TOF Nano HPLC-CHIP Cube Agilent 6520 instrument (Agilent Technologies, Santa Clara, CA, USA) using a linear gradient (0.4µl/min) from 0% solvent A (97% water/3% acetonitrile/0.1% formic acid) to 80% solvent B (97% acetonitrile/3% water/0.1% formic acid) in 10 minutes and from 80% to 5% solvent B in 5 minutes using a Zorbax C18 Column (43mm X 75µm, 5µm) equipped with an enrichment column (4mm, 40nl).

**Supplementary Figures**

**Supplementary Figure 1**

**
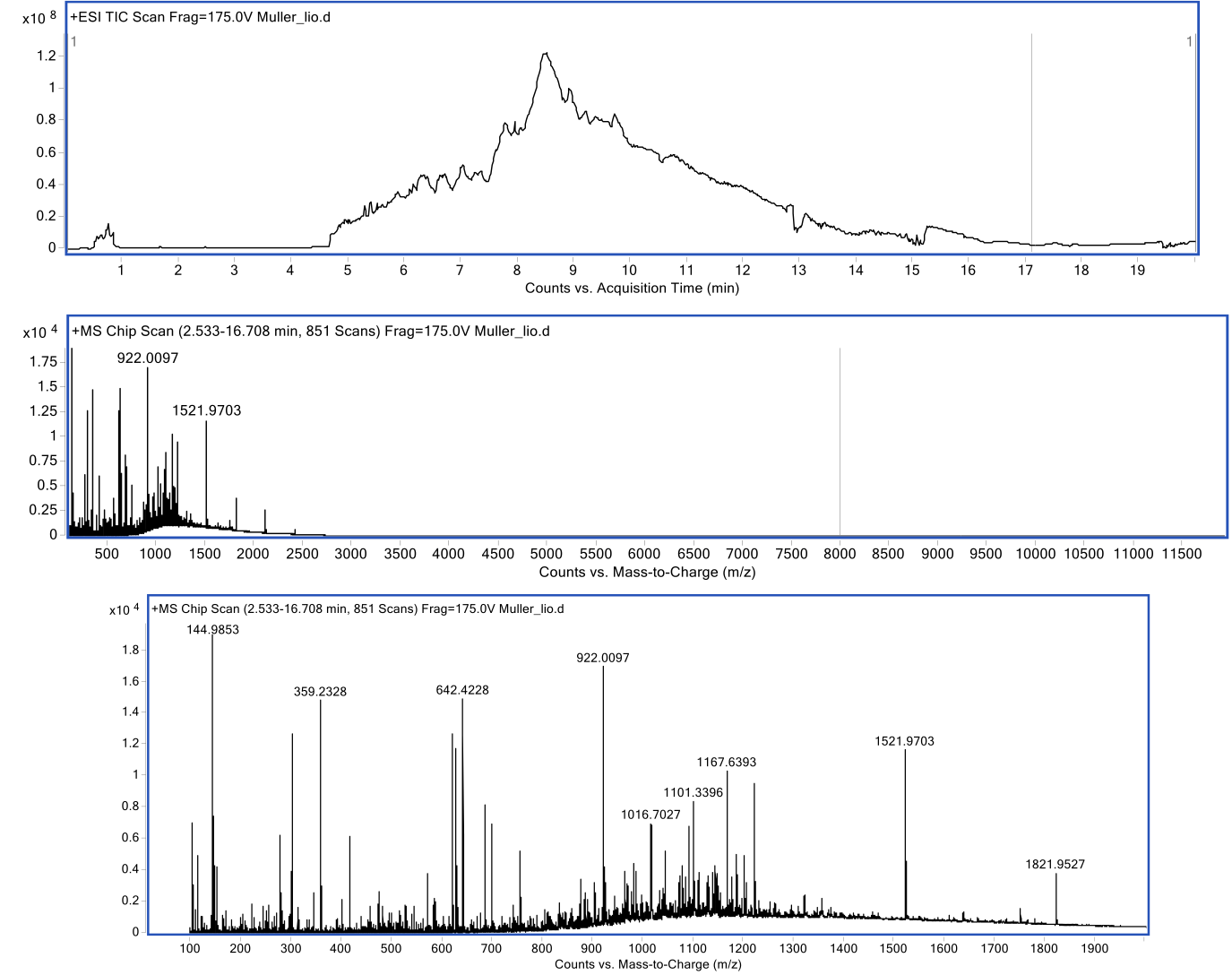
**

**Figure S1**: LC/MS analysis of the crude snail mucus extract showing a multiple complex peak.

**Supplementary Figure 2**


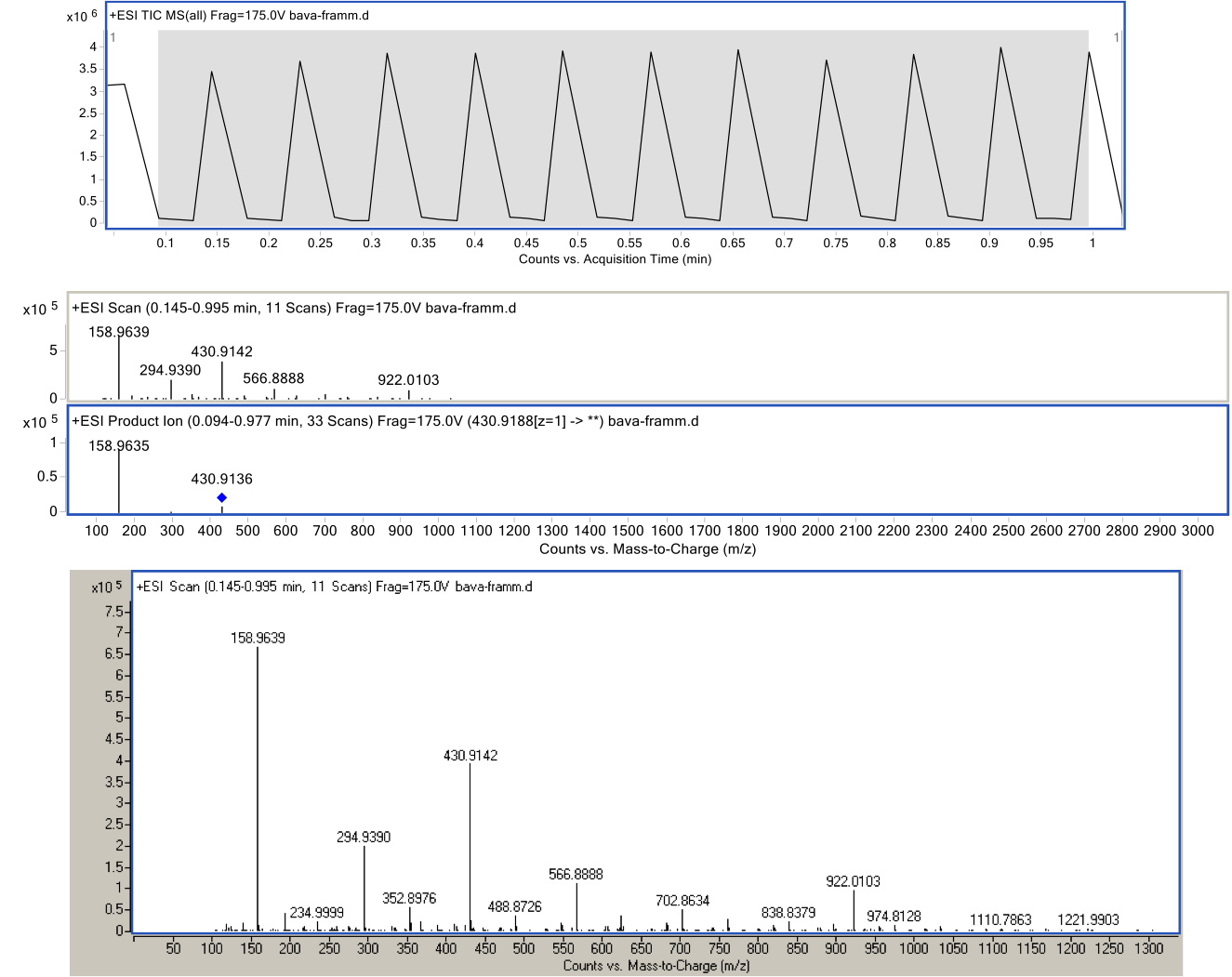


**Figure S2**: LC/MS/MS system analysis of the crude snail mucus focusing the fragmentation partners at the 430.9142 [M+H]^+^.
